# Reporting and Availability of COVID-19 Demographic Data by US Health Departments (April to October 2020): Observational Study

**DOI:** 10.2196/24288

**Published:** 2021-04-06

**Authors:** Peace Ossom-Williamson, Isaac Maximilian Williams, Kukhyoung Kim, Tiffany B Kindratt

**Affiliations:** 1 Research Data Services UTA Libraries University of Texas at Arlington Arlington, TX United States; 2 Public Health Program, Department of Kinesiology College of Nursing and Health Innovation University of Texas at Arlington Arlington, TX United States

**Keywords:** coronavirus disease 2019, COVID-19, SARS-CoV-2, race, ethnicity, age, sex, health equity, open data, dashboards

## Abstract

**Background:**

There is an urgent need for consistent collection of demographic data on COVID-19 morbidity and mortality and sharing it with the public in open and accessible ways. Due to the lack of consistency in data reporting during the initial spread of COVID-19, the Equitable Data Collection and Disclosure on COVID-19 Act was introduced into the Congress that mandates collection and reporting of demographic COVID-19 data on testing, treatments, and deaths by age, sex, race and ethnicity, primary language, socioeconomic status, disability, and county. To our knowledge, no studies have evaluated how COVID-19 demographic data have been collected before and after the introduction of this legislation.

**Objective:**

This study aimed to evaluate differences in reporting and public availability of COVID-19 demographic data by US state health departments and Washington, District of Columbia (DC) before (pre-Act), immediately after (post-Act), and 6 months after (6-month follow-up) the introduction of the Equitable Data Collection and Disclosure on COVID-19 Act in the Congress on April 21, 2020.

**Methods:**

We reviewed health department websites of all 50 US states and Washington, DC (N=51). We evaluated how each state reported age, sex, and race and ethnicity data for all confirmed COVID-19 cases and deaths and how they made this data available (ie, charts and tables only or combined with dashboards and machine-actionable downloadable formats) at the three timepoints.

**Results:**

We found statistically significant increases in the number of health departments reporting age-specific data for COVID-19 cases (*P*=.045) and resulting deaths (*P*=.002), sex-specific data for COVID-19 deaths (*P*=.003), and race- and ethnicity-specific data for confirmed cases (*P*=.003) and deaths (*P*=.005) post-Act and at the 6-month follow-up (*P*<.05 for all). The largest increases were race and ethnicity state data for confirmed cases (pre-Act: 18/51, 35%; post-Act: 31/51, 61%; 6-month follow-up: 46/51, 90%) and deaths due to COVID-19 (pre-Act: 13/51, 25%; post-Act: 25/51, 49%; and 6-month follow-up: 39/51, 76%). Although more health departments reported race and ethnicity data based on federal requirements (*P*<.001), over half (29/51, 56.9%) still did not report all racial and ethnic groups as per the Office of Management and Budget guidelines (pre-Act: 5/51, 10%; post-Act: 21/51, 41%; and 6-month follow-up: 27/51, 53%). The number of health departments that made COVID-19 data available for download significantly increased from 7 to 23 (*P*<.001) from our initial data collection (April 2020) to the 6-month follow-up, (October 2020).

**Conclusions:**

Although the increased demand for disaggregation has improved public reporting of demographics across health departments, an urgent need persists for the introduced legislation to be passed by the Congress for the US states to consistently collect and make characteristics of COVID-19 cases, deaths, and vaccinations available in order to allocate resources to mitigate disease spread.

## Introduction

The COVID-19 outbreak originated in December 2019 in China. On January 20, 2020, the Centers for Disease Control and Prevention (CDC) reported the first confirmed COVID-19 case in the United States in Snohomish County, Washington [1]. By March 2020, the USA had the highest number of reported cases and one of the highest test-positive rates globally. The USA was initially presented with a series of time-bound public health challenges during and immediately after that 3-month timespan, including implementing testing processes, engaging in timely data collection and reporting, and maintaining trust while educating the public. As of our last data collection period (October 24, 2020), there have been 8,469,976 confirmed cases and 223,393 deaths in the USA, with the highest number of newly confirmed cases reported on October 23, 2020 [2].

Since a national emergency was declared on March 13, 2020 [3], state and local health departments have not been provided the funding or resources to collect and make surveillance data on patient demographics, testing, hospitalizations, confirmed cases (morbidity), and mortality available for the general public, institutions and academic organizations to use for developing targeted risk communication efforts and prevention policies. The quick nature of the outbreak, in combination with a lack of clear guidelines as to what could or should be made publicly available led to staff at health departments working extended hours to determine what information can be shared while building the structure for regularly reporting COVID-19 data.

Meanwhile, interest in COVID-19 information resources skyrocketed. Major US news websites saw 50% growth in webpage visits, with coronavirus stories making up 10%-25% of pageviews [4,5]. In March 2020, English Wikipedia pageviews rose from 1.6 million to 146 million, primarily on pages about (1) COVID-19 and SARS-CoV-2 virus, (2) outbreak data related to particular regions, (3) celebrities and public figures who tested positive for COVID-19, and (4) other relevant pages discussing topics such as lockdowns and the socioeconomic impact of the pandemic [6].

Because the prevention of COVID-19 requires global participation in prevention efforts, with one infected person having the ability to lead to large clusters of infection, it is likely that confusing, missing, or otherwise inaccessible COVID-19 data acts as a *data void* [7,8] and contributes to the broader COVID-19 infodemic—or an increased spread of a disease due to growing misinformation on the internet that either fills in the gaps or outpaces trustworthy and reliable sources. A 17-year old launched an online COVID-19 tracker called ncov2019.live [9] in December 2019, while the available data were still scarce; the website garnered millions of visits internationally [10]. Citizens tend to lose confidence in public response due to gaps in how democracies communicate with the public, particularly when social distancing becomes voluntary and does not rely on state orders.

In response to this need, agencies and institutions have built dashboards based on official government reports and various newspapers sources. For instance, the COVID Tracking Project at The Atlantic [11] was built using official governmental reports, and the Johns Hopkins University COVID-19 Dashboard [12] was built using estimates based on various news and official reports. At their peaks, these dashboards saw over a billion visitors daily [11,12]. However, inconsistent reporting makes it difficult to accurately track and compare surveillance data on the pandemic across US states. For example, several popular sites (Google, Twitter, and Facebook) provide real-time information from the CDC or from data aggregate websites when users explore COVID-19–related topics. However, due to differences in reporting, data are typically limited to statewide or national case counts and rates [11,12]. Global comparisons are also difficult owing to challenges in reporting and making data publicly available. The World Health Organization (WHO) reports cumulative and newly reported confirmed COVID-19 cases and deaths by region on a weekly basis [13]. However, the definitions for confirmed cases or deaths differ by country, and the WHO does not publicly report or make available any additional data on sociodemographic characteristics to make between-country comparisons [14,15].

Barriers to data availability are numerous and include time and effort toward data synchronization and sharing; the need for sufficient platforms that facilitate sharing in ways that promote awareness but do not risk identification; and accuracy of metadata in addition to ethical, political, and legal boundaries (S. C. Clarke, MLIS, unpublished data, August 2019). Although case surveillance data collection and reporting internally to health departments tend to be executed in a well-structured format, these standards should be extended and translated into public reporting [16]. Having data available during disease outbreaks builds trust [17] and invokes the four ethical principles of social value, respect, justice, and transparency (S.C. Clarke, MLIS, unpublished data, August 2019). Data can be made available at individual-level, population-level, and exposure-level, each of which, if shared in measured ways with appropriate groups, is crucial for the prevention of sluggish response times and unnecessary suffering and death.

Throughout the initial spread of COVID-19 in the USA, reporting at the population-level (and exposure-level) was inconsistent, with many data points unreliable or unavailable [18]. Those that were provided were presented on health department websites in portable document format (PDF) files with file names that did not clearly express that the files contained incidence data. Moreover, there were issues with data being conflated as were the results of serology and virology tests on the websites of the CDC and various state health departments [19,20]. Demographic data were largely unavailable at all reporting levels, and, when present, incongruent reporting of age, sex, race, and ethnicity of cases and deaths contributed to the COVID-19 infodemic.

Therefore, not only is there an urgent need for a unified collection of demographic data on COVID-19 morbidity and mortality statistics, but data also need to be shared in open and accessible ways. Open and accessible data sharing allows for health educators, researchers, and lawmakers to calculate accurate statistics for implementing targeted interventions for specific states and develop policies to mitigate the spread and impact of the virus [21]. Integrating open and accessible data sharing also prevents issues of access that often come with charts and dashboards for those with visual impairment or cognitive disabilities (eg, dyslexia). The open data movement is a global attempt to make data freely available in a format that can be reused in machine-actionable downloadable formats by others [22]. Those opposed to providing open data highlight the significant number of resources needed to develop and maintain databases and concerns about legal and ethical issues with data sharing. However, there are several benefits to providing open data for public health that can directly improve data reporting and availability for the COVID-19 pandemic. For example, providing open data will allow for increased opportunity for early detection, improve real-time response, inform interventions and policy decisions, improve accountability, and enable transparency [22].

Due to mounting demands for transparency, on April 21, 2020, House of Representatives (HR) 6585, the Equitable Data Collection and Disclosure on COVID-19 Act was introduced into the Congress, with support from the American Public Health Association, to mandate the collection and reporting of demographic COVID-19 data on testing, treatment, and deaths by race, ethnicity, sex, age, disability, primary language, socioeconomic status, and county [23]. The bill states that the secretary of the US Department of Health and Human Services and the director of the CDC must make data publicly available on the CDC website. The bill recommends collecting data on race and ethnicity in line with other federal standards, including the Office of Management and Budget’s (OMB) guidelines for collecting data pertaining to race (White, Black or African American, Asian, American Indian or Alaskan Native, Native Hawaiian or Other Pacific Islander) and ethnicity (Hispanic or Latino, not Hispanic or Latino) [24]. Although this bill has not been passed as of the latest writing of this manuscript (January 20, 2021), its introduction laid the groundwork for greater exposure on the need for consistent demographic data linked with COVID-19 morbidity and mortality to be reported at the state level and be made openly accessible for the public research institutions, academic organizations, and the general public. There is a need to determine how its initial introduction into the Congress has impacted how data have been reported and made available by health departments during this rapidly changing pandemic.

To address this need, this study aimed to determine the consequence of a lack of standard reporting guidelines for US health departments. Our specific aims were to evaluate immediate and 6-month statewide differences in (1) reporting of demographic data for confirmed COVID-19 cases and deaths and (2) public availability of data by charts or tables only, dashboards, and machine-actionable datasets due to increasing public pressure for greater transparency.

## Methods

### Data Collection

We reviewed the websites of local health departments from all 50 states and Washington, District of Columbia (DC), to determine how COVID-19 data were made available to the general public, health educators, and researchers and to identify which demographic data were reported. In order to determine the immediate actions being taken in reporting and to determine changes in reporting over time, data were collected and reviewed before (April 13-20, 2020), immediately after (April 27-28, 2020), and 6 months after (October 23, 2020) the introduction of the Equitable Data Collection and Disclosure on COVID-19 Act on April 21, 2020.

### Data Reporting

We evaluated how each US state reported demographic characteristics of laboratory-confirmed COVID-19 cases and deaths. Variables were created to determine whether each state or municipality reported data on age (varying age groups), sex (including male and female), and race or ethnicity (none, race and ethnicity separated, or race and ethnicity as a combined measure) for confirmed COVID-19 cases and deaths. We also evaluated whether race and ethnicity data were reported based on federal reporting standards set for by the OMB, which includes the identification of White, Black or African American, Asian, American Indian or Alaskan Native, Native Hawaiian or Other Pacific Islander race and Hispanic or Latino ethnicity [24].

### Data Availability

Data availability is described as “the degree of convenience for users to obtain data and related information….[and] includes the difficulty level that users may experience when accessing data” and its timeliness [25]. A data quality indicator should incorporate existence of a specific data component, data availability at a specific geographical scale, relevance to the users’ needs and “data quality components that are used to build a composite index in which indicator quality is assessed under a scoring system” [25]. As a result, we created a data availability score to compare how each US state made data available to the public, to researchers, journalists, and public health professionals. The frequency of reporting was not analyzed, as states were reporting new data daily despite technical difficulties. A Likert scale of 1 to 4 was developed, with the following options: 1=totals only (COVID-19 cases and deaths); 2=charts and figures showing disaggregation of any kind; 3= interactive dashboards; and 4=machine-actionable data (ie, data downloadable in a format readable by data analytics software).

### Statistical Analysis

Descriptive statistics (frequencies and percentages) were used to present data reporting characteristics by each state in tabular and visual formats. McNemar tests were used to determine significant (*P*<.05) changes in COVID-19 morbidity and mortality data reporting and availability by health departments before and immediately after as well as before and six months after the introduction of the Equitable Data Collection and Disclosure on COVID-19 Act on April 21, 2020. Analyses were conducted using STATA 14.0 (StataCorp LLC) [26].

This secondary study did not involve human subjects; therefore, institutional review board approval was not required.

## Results

**Figure 1:**
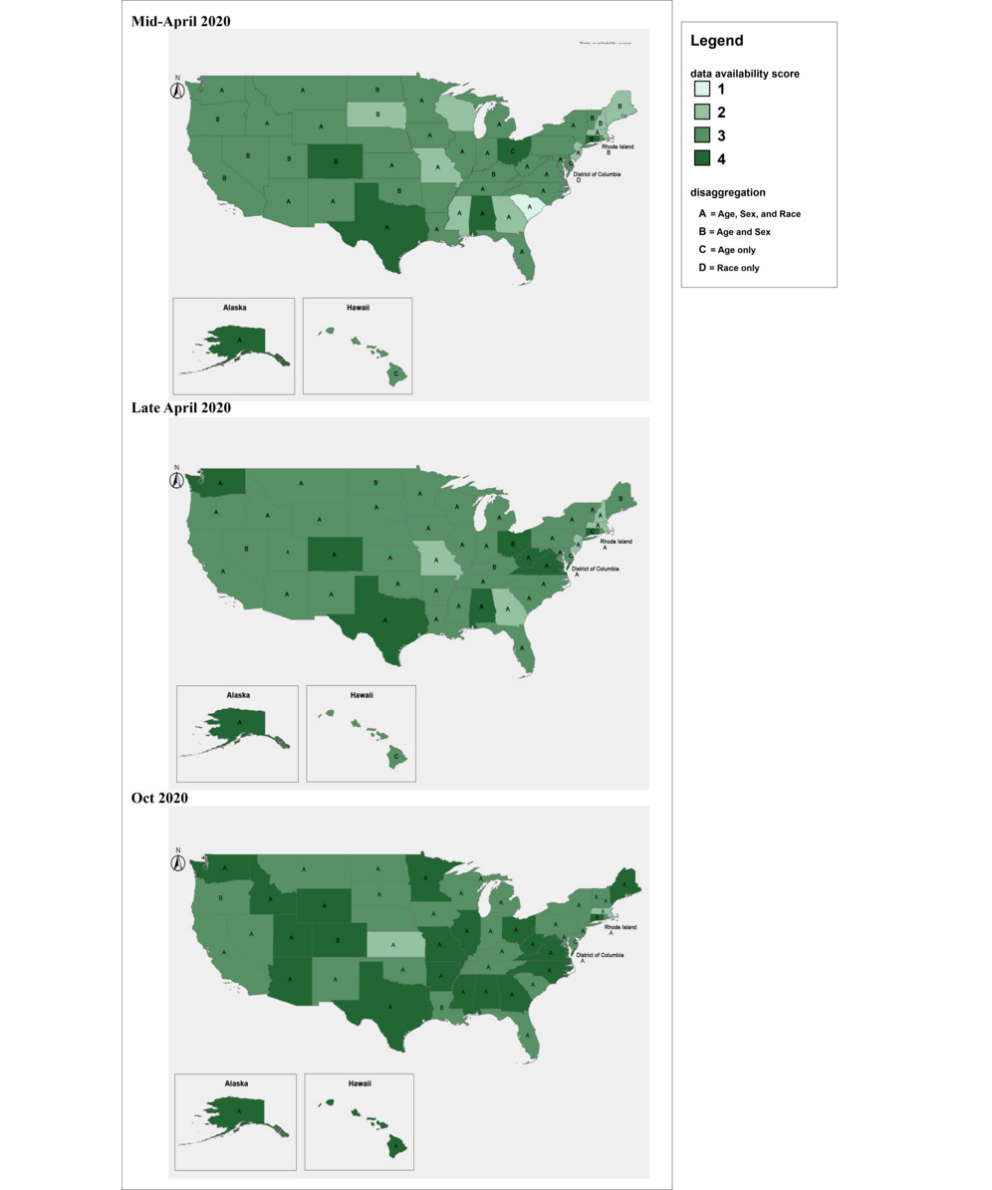
Demographic COVID-19 data reported by state public health departments of the Unites States (A) before, (B) immediately after, and (C) 6 months after the introduction of the Equitable Data Collection and Disclosure on COVID-19 Act. Data availability scores: 1=totals only (confirmed cases and deaths in the state); 2=figures or web tables showing disaggregation of any kind; 3=interactive dashboards; and 4=machine-actionable data. Disaggregation types available: A=ASR (age, sex, race), B=AS (age and sex), C=A (age), D=R (race).

The data availability scores for each state and how data were reported before and immediately after the Equitable Data Collection and Disclosure on COVID-19 Act was introduced into the Congress are illustrated in Figure 1.

Statistical comparisons of how data were reported for confirmed COVID-19 cases and deaths and made available to the public before, immediately after, and 6 months after the introduction of the Equitable Data Collection and Disclosure on COVID-19 Act are presented in Table 1. From April 13 to 20, 2020, 46 of 51 (90.2%) health departments reported age-specific data for confirmed COVID-19 cases but only 25 of 51 (49%) health departments reported age-specific data for patients who died due to COVID-19. There was a statistically significant increase in the number of health departments that reported confirmed COVID-19 cases immediately after and 6 months after the act was introduced (50/51, 98%, *P*=.045) and COVID-19 deaths by age immediately after the act was introduced (35/51, 69%, *P*=.002) and 6 months later (39/51, 76%, *P*=<.001). Despite these significant increases, differences still remained between states in the ways that age was reported. For example, the health department of California reports the following age groups for confirmed cases and deaths: 0-17 years, 18-49 years, 50-64 years, and 65 years and older [27]. The health department of Texas reports the age groups for COVID-19 cases and resulting deaths across multiple categories: <1 year, 1-9 years, 10-19 years, 20-29 years, 30-39 years, 40-49 years, 50-59 years, 60-64 years, 65-69 years, 70-74 years, 75-79 years, and 80 years and older [28]. From April 13 to 20, 2020, only 21 of 51 (41%) health departments reported the sex of COVID-19 deaths. This number rose after the introduction of the legislation (30/51, 59%, *P*=.003) and 6 months later (36/51, 71%, *P*<.001). Significant increases were found in the number of health departments reporting the race or ethnicity for confirmed cases and deaths (*P*<.001) immediately after (*P*<.001 confirmed cases; *P*<.001 deaths) and 6 months after (both *P*<.001) the introduction of the legislation on April 21, 2020. Moreover, the number of health departments that reported race or ethnicity data using federal standards more than quadrupled over the 6-month data collection period, that is, from 10% (5/51) to 53% (27/51; *P*<.001). Prior to the introduction of the legislation, a majority (35/51, 69%) of state health departments provided COVID-19 surveillance data using dashboards, but only 7 (14%) of 51 health departments provided machine-actionable data that could be downloaded and used for additional reporting and analyses by public health researchers. Six months later, 23 (45%) of 51 health departments made their data available to the public for download (*P*<.001).

**Table 1:** Statistical comparisons of data reported before (pre-Act), immediately after (post-Act), and 6 months after (6-month follow-up) the introduction of the Equitable Data Collection and Disclosure on COVID-19 Act (N=51).

Data reported		Value, n (%)				*P* value^b^
		Pre-Act	Post-Act	6-month follow-up		
**Age^a^**						
	Confirmed cases	46 (90)	50 (98)	50 (98)	.045	
	Deaths	25 (49)	35 (69)	39 (77)	<.001	
**Sex^a^**						
	Confirmed cases	45 (88)	48 (94)	49 (96)	.045	
	Deaths	21 (41)	30 (57)	36 (71)	<.001	
**Race and ethnicity^a^**						
	Confirmed cases	18 (35)	31 (61)	46 (90)	<.001	
	Deaths	13 (24)	25 (49)	39 (77)	<.001	
OMB^c^ standards		5 (10)	22 (43)	27 (53)	<.001	
**Data availability**						
	Charts or tables only	9 (18)	9 (18)	2 (4)	<.001	
	Dashboards^d^	35 (69)	31 (61)	26 (51)	<.001	
	Machine-actionable^e^	7 (14)	11 (22)	23 (45)	<.001	

^a^Data collection for age, sex, and race and ethnicity for confirmed COVID-19 cases and deaths took place during April 13-20, April 23-27, and October 23, 2020.

^b^McNemar test results reported for pre-Act and 6 months post-Act comparisons.

^c^OMB: Office of Management and Budget.

^d^Dashboards in addition to charts and tables.

^e^Machine-actionable data in addition to charts and tables.

## Discussion

### Principal Findings

The purpose of our study was to describe and compare how US state and Washington, DC, health departments reported COVID-19 confirmed cases and deaths and made data available to the public. Demographic characteristics and data availability were analyzed and compared before, immediately after, and 6 months after the legislation (Equitable Data Collection and Disclosure on COVID-19 Act on April 21, 2020) was proposed, urging consistent collection and reporting of certain COVID-19 data [24]. Reporting of data based on race and ethnicity showed the greatest increase over the given time period. Three main findings were observed.

First*,* we found a significant increase in the number of states reporting COVID-19 surveillance data disaggregated by race and ethnicity during our study period. In addition to the presentation of a new legislation, this is likely due to the growing media coverage spotlighting this gap in reporting [29]. Illinois became one of the first US states to report race and ethnicity data on confirmed COVID-19 cases on March 26, 2020 [30]. Comparable data from the state of Connecticut are the only estimates published in the medical literature thus far [31]. Although we found that a significantly higher number of states reported racial and ethnic breakdowns based on federal standards, only half of them (27/51, 53%) reported it using the OMB guidelines. The racial and ethnic composition of the US population has significantly changed since the federal standards were implemented in 1997, and within-group differences (eg, West African Black, Southeast Asian, or Arab White) were not available using these categorizations. Nonetheless, there is a need for consistent reporting and availability of racial and ethnic data across states to use as a baseline that can be expanded to identify disparities in racial and ethnic subgroups. Although states have made progress in the collection of racial and ethnic data for confirmed COVID-19 cases, hospitalizations, and deaths among populations that are non-Hispanic Black or African American, Hispanic or Latino, and Asian, smaller groups such as Native Hawaiian and Pacific Islander populations are often neglected or categorized as “other” despite the OMB guidelines [32]. As of August 13, 2020, only 20 (39%) of 51 health departments reported Native Hawaiian and Pacific Islander estimates based on federal regulations. There is an urgent need for US states to collect and report fully representative COVID-19 morbidity and mortality data by race and ethnicity so that resources can be allocated and policy decisions can be improved for minority groups disproportionately affected by COVID-19.

Second*,* we found a significant increase in the number of states reporting COVID-19 surveillance data based on age. With age being one of the first identified risk factors for COVID-19 complications, it is surprising that this has not been reported consistently for confirmed cases and deaths since the start of the COVID-19 pandemic. Despite the need for reporting this data, researchers have cautioned that emphasizing on chronological age can be detrimental to older adults. In a recent editorial, Ayalon and colleagues [33] highlighted that the focus on reporting COVID-19 case data by chronological age only can lead to a parallel epidemic of discrimination, increasing societal age divisions between the young and old, and ethical challenges among overburdened health care systems. Nonetheless, media reports have consistently focused on the need for older adults to stay at home and for younger individuals to contribute to social distancing in order to protect older adults [33]. Studies are underway to determine the beneficial and detrimental effects of social distancing by measuring changes in loneliness, communication, and physical contact among close family and friends before and after social distancing measures were established [34]. As states move to report more accurate data on age, researchers on aging caution against reporting chronological age as a risk factor using arbitrary cutoffs, but in conjunction with other factors such as chronic illness and comorbidities. In this study, we did not systematically collect data on the number of states that reported pre-existing conditions of confirmed COVID-19 cases and deaths in, and this data has been rarely collected and reported in the context of age of patients. If the Equitable Data Collection and Disclosure on COVID-19 Act is passed by the Congress, the CDC will be required to report this information [23]. If this data is made openly available and accessible, further clarification of risks among individuals of all ages can be more accurately explained.

Third, we found a significant increase in the number of states that provided machine-actionable data immediately after and 6 months after the introduction of the legislation. Most states (49/51, 96%) had interactive dashboards available, whereas only 23 states and Washington, DC, (45%) had machine-readable morbidity and mortality data available (in addition to their dashboards). Dashboards were created through data visualization tools and geographic information system software and have become a modern way to present basic epidemiologic data that tracks disease by location, which has been used for centuries. The most well-known dashboard that presents US COVID-19 data is not from a public health department, but from Johns Hopkins University [35]. The dashboard was developed and first made available to the public on January 22, 2020; it has since received billions of views and shares on social media platforms and has been recognized by several media outlets. In March 2020, with 4.56 billion feature requests, the Johns Hopkins University COVID-19 Dashboard outpaced Pokémon Go, the mapping software by Esri’s formerly most popular map [36]. An initial limitation of this data source was its inability to provide this information at the local level in combination with national and international surveillance. However, county-level data are now available [37]. Despite the value that dashboards provide to the public for tracking and mitigating the spread of disease, there is a need to go beyond visualizations and provide open data for researchers and policy makers to fully capture health disparities in real time. As previously mentioned, if the Equitable Data Collection and Disclosure on COVID-19 Act is passed, the CDC will be required to collect and provide this information [23]. If the infrastructure is not put in place for the CDC to collect this information in a unified manner, there will be a need for other national surveys such as the National Health Interview Survey collected by the National Center Health Statistics to be revised to include variables to identify these health indicators.

In addition to the three main findings described above, this study revealed other areas that are currently lacking in reporting of data. The majority of data reported are still being provided as *counts*. In addition to the need for disaggregation, states are far from data reporting using key epidemiological concepts such as population attack rate, case fatality rate, and models of COVID-19 incidence that include estimates of untested cases; these data points are necessary to enable policy decisions [21]. More advanced methods of reporting are also needed to make more meaningful comparisons between states and at the international scale. Differences in reporting data on COVID-19 confirmed cases and deaths across countries have made standard epidemiological comparisons more difficult. For example, Belgium includes all deaths that have COVID-19 suspected as a contributing cause, translating to considerably higher mortality rates than those reported by other countries [14]. Countries without widespread testing for COVID-19 may also report higher mortality rates due to smaller denominators of total confirmed cases, yet lower overall case fatality rates. Differences in case fatality rates may also be due to national differences in public health infrastructure, policy interventions, comorbidities, and sociodemographic factors [15]. Calculating excess all-cause mortality during the COVID-19 pandemic may be a more comprehensive way of making comparisons of the impact of COVID-19 on deaths both within and between countries [38].

At the end of our data collection period, only Hawaii, Indiana, Kansas, North Carolina, and Utah reported race or ethnicity rates to population (5/51, 10%), and only the state health department of California reported race and ethnicity data on confirmed COVID-19 cases and deaths in comparison with the corresponding percentages of the population [39]. A recent report by Resolve to Save Lives, an initiative endorsed by Trust for America’s Health, the American Public Health Association, Association of Schools & Programs of Public Health, and Johns Hopkins Bloomberg School of Public Health, developed 15 essential indicators representing 780 data points stratified by sex, age, and race and ethnicity as best practices for collecting and presenting data on COVID-19 surveillance dashboards. As of July 2020, at least 60% of the recommended data points were not reported by dashboards in some way [40].

### Limitations

Although our pre-post analysis showed significant improvements in state-level data reporting before and after the introduction of the Equitable Data Collection and Disclosure on COVID-19 Act, our results do not account for other factors, such as media coverage and improved knowledge of the disease. Therefore, we recognize the significant improvements may not directly represent causality. The biggest limitation of this study was information bias due to the rapid changes in reporting of surveillance data on a daily basis. States reported different case and death counts for each day, but case counts often increased, sometimes dramatically, and decreased at times, due to new count standards, data maintenance, and weekends and holidays. There was also variability in the ways the states reported demographics. For example, although several states reported age, sex, and race and ethnicity data from April 13-20, 2020, the way the data were reported varied between cases and deaths. The Equitable Data Collection and Disclosure on COVID-19 Act calls for morbidity and mortality surveillance data to be collected by sex, age, race and ethnicity, primary language, socioeconomic status, disability, and county [23]. Our study was limited to differences by sex, age, and race and ethnicity. However, we hope to explore how other data on primary language, socioeconomic status, chronic disease, and disability are reported and made available in future studies.

### Conclusions

The variation in reporting practices at both state and local levels indicate a need for standardized reporting across the US and for a national infrastructure for monitoring, auditing, and evaluating the quality of data reporting by states and subregions. The improvement in data reporting can be attributed to several factors, including increased clarity and capability of sharing data along with public demand for improved reporting; however, the need continues and includes additional stratification to provide more data points, including hospitalizations and hospital availability as well as COVID-19 vaccine availability and rates of vaccination among populations.

Despite numerous barriers to data sharing—which include technical, motivational, political, economic, legal, and ethical barriers (S. C. Clarke, MLIS, unpublished data, August 2019), policy makers must set reporting requirements for local public health agencies to determine what data should be made publicly available and how the data should be communicated to the public. Furthermore, in line with the open data movement, if at all possible, data should be shared in machine-actionable formats, allowing others to explore the data for spotting new trends and early detection, informing decision-making, and ensuring transparency and accountability.

Having standardized methods of counting, calculating, and reporting incidence can prevent future disagreements about data accuracy [41]. Two areas of particular importance are rates, rather than solely raw numbers, of confirmed cases and deaths provided by complete and consistent age, race, and ethnicity desegregation, as this can guide public response and resource allocation, especially as vaccinations become available for an increasing number of people. As vaccines are distributed and administered using different criteria and with differing uptake in various populations, websites reporting COVID-19 data and progress continue to be exceedingly useful as a primary source for the public to continually stay informed. Moreover, there is an urgent need for the legislation to be passed for all US states to consistently collect and make characteristic data of confirmed COVID-19 cases and deaths publicly available in order to allocate resources to mitigate the spread of the disease.

## Abbreviations

CDC: Centers for Disease Control and Prevention
DC: District of Columbia
HR: House of Representatives
OMB: Office of Management and Budget
PDF: portable document format

## References

[1] Holshue ML, DeBolt C, Lindquist S, Lofy KH, Wiesman J, Bruce H, Spitters C, Ericson K, Wilkerson S, Tural A, Diaz G, Cohn A, Fox L, Patel A, Gerber SI, Kim L, Tong S, Lu X, Lindstrom S, Pallansch MA, Weldon WC, Biggs HM, Uyeki TM, Pillai SK, Washington State 2019-nCoV Case Investigation Team (2020). First case of 2019 novel coronavirus in the United States. N Engl J Med.

[2] COVID Data Tracker. Centers for Disease Control and Prevention.

[3] (2020). Proclamation on Declaring a National Emergency Concerning the Novel Coronavirus Disease (COVID-19) Outbreak. The White House.

[4] Benton J (2020). The coronavirus traffic bump to news sites is pretty much over already. NiemenLab.

[5] Molla R (2020). It’s not just you. Everybody is reading the news more because of coronavirus. Vox.

[6] Sáez-Trumper D (2020). Open data and COVID-19: Wikipedia as an informational resource during the pandemic. Medium.

[7] Shu Catherine (2019). Pinterest starts displaying information from health organizations for searches related to vaccines. TechCrunch.

[8] Gynes N, Mina AX (2020). How Misinfodemics Spread Disease. The Atlantic.

[9] World COVID-19 Stats. ncov2019.live.

[10] Bazzaz D (2020). The Seattle Times.

[11] About Us. The COVID Tracking Project.

[12] COVID-19 Map FAQ. Johns Hopkins Coronavirus Resource Center.

[13] WHO Coronavirus Disease (COVID-19) Dashboard. World Health Organization.

[14] Balmford B, Annan JD, Hargreaves JC, Altoè M, Bateman IJ (2020). Cross-country comparisons of Covid-19: policy, politics and the price of life. Environ Resour Econ (Dordr).

[15] Sorci G, Faivre B, Morand S (2020). Explaining among-country variation in COVID-19 case fatality rate. Sci Rep.

[16] Choi H, Cho W, Kim M, Hur J (2020). Public health emergency and crisis management: case study of SARS-CoV-2 outbreak. Int J Environ Res Public Health.

[17] Glik DC (2007). Risk communication for public health emergencies. Annu Rev Public Health.

[18] COVID-19 case-tracking website abruptly pulled. Riverside/Brookfield Landmark.

[19] Stolberg Sheryl, Kaplan S, Mervosh S COVID-19 case-tracking website abruptly pulled. The New York Times.

[20] Meyer R, Madrigal AC (2020). State and Federal Data on COVID-19 Testing Don’t Match Up. The Atlantic.

[21] Pearce N, Vandenbroucke JP, VanderWeele TJ, Greenland S (2020). Accurate statistics on COVID-19 are essential for policy guidance and decisions. Am J Public Health.

[22] Huston P, Edge VL, Bernier E (2019). Reaping the benefits of open data in public health. Can Commun Dis Rep.

[23] H.R.6585 - Equitable Data Collection and Disclosure on COVID-19 Act 116th Congress (2019-2020). Congress.gov.

[24] (1997). Revisions to the Standards for the Classification of Federal Data on Race and Ethnicity. The White House.

[25] Costa C, Stefanik I, Krafft T, Pilot E, Morrison J, Santana P, Freitas (2019). Evaluation of data availability on population health indicators at the regional level across the European Union. Popul Health Metr.

[26] (2015). Stata Statistical Software: Release 14. StataCorp.

[27] Tracking COVID-19 in California. State of California - COVID19.ca.gov.

[28] COVID-19 In Texas (Dashboard). Texas Health and Human Services.

[29] Kendi IX (2020). Why Don’t We Know Who the Coronavirus Victims Are?. The Atlantic.

[30] Public Health Officials Announce 673 New Confirmed Cases of Coronavirus Disease. Illinois Department of Public Health.

[31] Laurencin CT, McClinton A (2020). The COVID-19 pandemic: a call to action to identify and address racial and ethnic disparities. J Racial Ethn Health Disparities.

[32] Chang RC, Penaia C, Thomas K (2020). Count Native Hawaiian And Pacific Islanders In COVID-19 Data—It’s An OMB Mandate. Health Affairs.

[33] Ayalon L, Chasteen A, Diehl M, Levy B, Neupert SD, Rothermund K, Tesch-Römer C, Wahl H (2021). Aging in times of the COVID-19 pandemic: avoiding ageism and fostering intergenerational solidarity. J Gerontol B Psychol Sci Soc Sci.

[34] Cawthon P, Orwoll E, Ensrud K, Cauley JA, Kritchevsky SB, Cummings SR, Newman A (2020). Assessing the impact of the COVID-19 pandemic and accompanying mitigation efforts on older adults. J Gerontol A Biol Sci Med Sci.

[35] Kamel Boulos MN, Geraghty EM (2020). Geographical tracking and mapping of coronavirus disease COVID-19/severe acute respiratory syndrome coronavirus 2 (SARS-CoV-2) epidemic and associated events around the world: how 21st century GIS technologies are supporting the global fight against outbreaks and epidemics. Int J Health Geogr.

[36] Pearce K (2020). The unsung mapmakers. The Hub.

[37] John Hopkins Coronavirus Resource Center.

[38] Bilinski A, Emanuel EJ (2020). COVID-19 and excess all-cause mortality in the US and 18 comparison countries. JAMA.

[39] (2020). COVID-19 Race and Ethnicity Data. California Department of Public Health.

[40] Tracking COVID-19 in the United States. Prevent Epidemics.

[41] Wallman B (2020). Analyst was praised for creating and running Florida’s coronavirus data website. Now she says she was fired for challenging secrecy. South Florida Sun Sentinel.

